# Highly accelerated 4D flow cardiovascular magnetic resonance using a pseudo-spiral Cartesian acquisition and compressed sensing reconstruction for carotid flow and wall shear stress

**DOI:** 10.1186/s12968-019-0582-z

**Published:** 2020-01-20

**Authors:** Eva S. Peper, Lukas M. Gottwald, Qinwei Zhang, Bram F. Coolen, Pim van Ooij, Aart J. Nederveen, Gustav J. Strijkers

**Affiliations:** 1 Department of Radiology and Nuclear Medicine, Amsterdam UMC, University of Amsterdam, Amsterdam, the Netherlands; 2 Department of Biomedical Engineering and Physics, Amsterdam UMC, University of Amsterdam, Amsterdam, the Netherlands

**Keywords:** Compressed sensing, 4D flow MRI, Undersampling, Pseudo-spiral sampling, Cartesian, Incoherent sampling, Total variation, Acceleration

## Abstract

**Background:**

4D flow cardiovascular magnetic resonance (CMR) enables visualization of complex blood flow and quantification of biomarkers for vessel wall disease, such as wall shear stress (WSS). Because of the inherently long acquisition times, many efforts have been made to accelerate 4D flow acquisitions, however, no detailed analysis has been made on the effect of Cartesian compressed sensing accelerated 4D flow CMR at different undersampling rates on quantitative flow parameters and WSS.

**Methods:**

We implemented a retrospectively triggered 4D flow CMR acquisition with pseudo-spiral Cartesian k-space filling, which results in incoherent undersampling of k-t space. Additionally, this strategy leads to small jumps in k-space thereby minimizing eddy current related artifacts. The pseudo-spirals were rotated in a tiny golden-angle fashion, which provides optimal incoherence and a variable density sampling pattern with a fully sampled center. We evaluated this 4D flow protocol in a carotid flow phantom with accelerations of R = 2–20, as well as in carotids of 7 healthy subjects (27 ± 2 years, 4 male) for R = 10–30. Fully sampled 2D flow CMR served as a flow reference. Arteries were manually segmented and registered to enable voxel-wise comparisons of both velocity and WSS using a Bland-Altman analysis.

**Results:**

Magnitude images, velocity images, and pathline reconstructions from phantom and in vivo scans were similar for all accelerations. For the phantom data, mean differences at peak systole for the entire vessel volume in comparison to R = 2 ranged from − 2.3 to − 5.3% (WSS) and − 2.4 to − 2.2% (velocity) for acceleration factors R = 4–20. For the in vivo data, mean differences for the entire vessel volume at peak systole in comparison to R = 10 were − 9.9, − 13.4, and − 16.9% (WSS) and − 8.4, − 10.8, and − 14.0% (velocity), for R = 20, 25, and 30, respectively. Compared to single slice 2D flow CMR acquisitions, peak systolic flow rates of the phantom showed no differences, whereas peak systolic flow rates in the carotid artery in vivo became increasingly underestimated with increasing acceleration.

**Conclusion:**

Acquisition of 4D flow CMR of the carotid arteries can be highly accelerated by pseudo-spiral k-space sampling and compressed sensing reconstruction, with consistent data quality facilitating velocity pathline reconstructions, as well as quantitative flow rate and WSS estimations. At an acceleration factor of R = 20 the underestimation of peak velocity and peak WSS was acceptable (< 10%) in comparison to an R = 10 accelerated 4D flow CMR reference scan. Peak flow rates were underestimated in comparison with 2D flow CMR and decreased systematically with higher acceleration factors.

## Background

3D phase contrast cine cardiovascular magnetic resonance (CMR) (4D flow CMR) [1] may help in risk stratification and follow-up of cardiovascular diseases, which is the leading cause of death worldwide [2]. Atherosclerosis is characterized by the accumulation of fatty deposits in the arterial walls, leading to inflammation, wall thickening, and reduced arterial elasticity [3–5] and is a main cause of cardiovascular disease. In relation to that, low wall shear stress (WSS), the tangential force of the blood flow on the vessel wall [6–8], was found to be an indicator for rearrangement of endothelial cells [9] and increased wall thickness [10]. Since atherosclerosis is a chronic disease that can stay asymptomatic for a long time, a great need exists for diagnostic tools that identify patients at high risk and at an early stage of the disease.

4D flow CMR enables comprehensive visualization and quantification of complex blood flow and the calculation of several relevant hemodynamic biomarkers, including WSS. However, 4D flow CMR acquisitions in general still remain challenging, mostly because of the long acquisition times involved. These can vary between 15 and 40 min [11–13], depending on the desired 3D spatial resolution, number of frames per cardiac cycle, the heart rate, the respiratory gating efficiency and (if applied) the use of acceleration techniques, such as sensitivity encoding (SENSE) or segmented k-space acquisitions. Overall, this seriously complicates its use in routine clinical practice, since apart from practical issues such as long waiting times and costs, it is difficult for patients to remain motionless in the scanner for such a long time. Also, physiological variations and movement during long scans may lead to artifacts and inaccuracies. Therefore, a clear need exists for scan time reduction in 4D flow CMR.

Various methods have been presented to accelerate 4D flow CMR [12, 14–17], which are commonly based on undersampling k-space (e.g. acquiring a reduced amount of data at a sub-Nyquist rate) and performing reconstructions at full image quality using advanced reconstruction algorithms. 4D flow CMR and other time-resolved cardiac imaging techniques are highly correlated in time. Therefore, these data are very suitable for applying undersampling, particularly in the time dimension. The use of non-Cartesian radial [14], spiral [15] and cone-shaped [16] undersampling techniques has been demonstrated, enabling highly accelerated and motion-robust scans. Nevertheless, non-Cartesian scans can be challenging with respect to implementation, post-processing, and reconstruction time. Cartesian k-t undersampling techniques, such as k-t SENSE [18] or k-t PCA [12] have shown to be successful with acceleration factors up to R = 8. EPI accelerated 4D flow CMR scans provided acceleration factors of R = 5 [19]. More recently, compressed sensing (CS) accelerated 4D flow techniques have been introduced, which facilitated even higher acceleration factors (R = 8–27) [17].

However, no comprehensive evaluation has been made on how highly (prospectively) Cartesian undersampling with CS reconstruction in 4D flow CMR affects quantitative flow parameter estimates like WSS in vivo.

For this study, we therefore aimed to firstly implement a highly accelerated 4D flow acquisition based on a Cartesian pseudo-spiral sampling trajectory. This resulted in incoherent undersampling patterns for each individual cardiac frame, enabling CS reconstructions using temporal constraints. We secondly evaluated the performance of this CS 4D flow CMR technique over a range of acceleration factors (R = 2–30) for estimating quantitative flow and WSS parameters in a carotid flow phantom, as well as in the carotid arteries of healthy subjects.

## Methods

### Sampling strategy and implementation

**Fig. 1:**
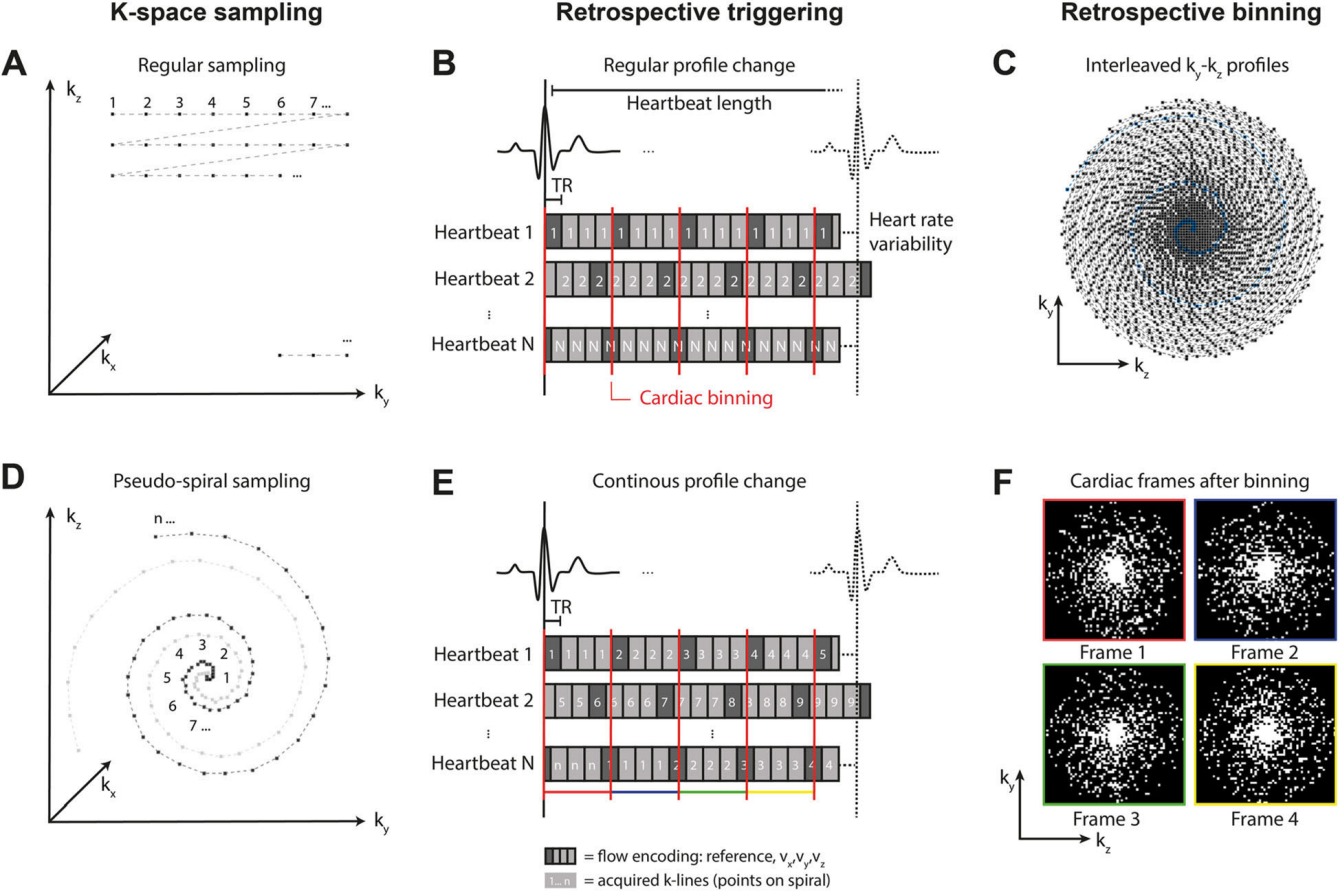
The pseudo-spiral sampling strategy for Cartesian 3D k-space sampling, with two phase encoding directions (k_y_,k_z_) and one fully sampled frequency encoding direction (k_x_). **a** In a regular Cartesian sampling strategy (k_y_,k_z_)-profiles are acquired line-by-line. **b** Additionally, in regular retrospectively triggered cardiac scans, each (k_y_,k_z_)-profile is repeated within each cardiac cycle to ensure complete filling of k-t-space and a fully sampled time dimension for each (k_y_,k_z_)-profile. The segments of 4xTR length indicate all 4 flow encoding segments of a 4D flow CMR scan, with the dark segments indicating the reference scan. **c** This distribution shows all pseudo-spiral (k_y_,k_z_)-coordinates sampled within one scan session. The pseudo-spiral trajectory creates a variable density distribution with an oversampled center. **d** In a pseudo-spiral sampling strategy, (k_y_,k_z_)-profiles are sampled from the center of k-space to the outside, however still on a Cartesian grid. **e** To achieve undersampling in the temporal domain, (k_y_,k_z_)-profiles are updated continuously within each cardiac cycle in addition to the pseudo-spiral sampling trajectory. **f** After retrospective cardiac binning, this results in a sampling mask of white (sampled) and black (not sampled) (k_y_,k_z_)-coordinates, with unique, incoherent sampling patterns per cardiac frames (see color coding in **e**)

Our specific method of accelerating 4D flow CMR using pseudo-spiral Cartesian sampling is schematically depicted in Fig. 1. 3D k-space consists of two phase encoding directions (k_y_,k_z_) and one fully sampled frequency encoding (readout) direction (k_x_). In regular Cartesian sampling (k_y_,k_z_)-profiles (a combination of k_y_ and k_z_ coordinates) would be acquired line-by-line (Fig. 1a). Additionally, in traditional cine imaging each (k_y_,k_z_)-profile would be sampled multiple times during each heartbeat (regular profile change) to ensure complete filling of k-t-space and a fully sampled time dimension for each (k_y_,k_z_)-profile (Fig. 1b). However, highly undersampling k-space in a random order on a Cartesian grid is not preferred since it would involve large steps in k-space. Regular line-by-line undersampling would complicate interleaving k-space sampling of multiple cardiac frames.

We implemented a pseudo-spiral filling strategy which was proposed earlier by Liu and Saloner [20] and also used in a similar fashion in G-CASPR [21], VDRad [17], ROCK-MUSIC [22] and GOCART [23] to create a randomly undersampled k-t-space. The pseudo-spiral trajectory has a couple of advantages. First, it involves only small jumps in k-space thereby minimizing eddy current related artifacts. Secondly, the pseudo-spirals can be rotated in a tiny golden-angle fashion, which provides optimal incoherence and a variable density sampling pattern with a fully sampled center (Fig. 1c), which is highly beneficial for CS reconstruction. Finally, despite the pseudo-spiral acquisition, the k-space points are still located on a Cartesian grid (hence the name pseudo-spiral), as shown in Fig. 1d, which improves the reconstruction as no regridding interpolation is required.

The second important mechanism in our approach is related to the cardiac triggering strategy (Figs. 1e, f). By using retrospective gating and a continuous update of the (k_y_,k_z_)-profile throughout the cardiac cycle, each separate cardiac frame will have its unique random k-space filling, but with preservation of the variable density pattern. This maximizes randomness in both the spatial and temporal (i.e. cardiac frames) domain. Note that first all four flow-encoding directions are sampled (4xTR) before changing the (k_y_,k_z_)-profile number.

The pseudo-spiral sampling trajectory in (k_y_,k_z_)-plane can be described in more detail as a spiral with radius r and linearly increasing angle φ gridded on the scan matrix size:

1 $$ \mathrm{r}\left(\upvarphi \right)={\upvarphi}^2\ \mathrm{with}\ \upvarphi \in \left\{{\upvarphi}_1=0,..,{\upvarphi}_{\mathrm{n}}=2\uppi \mathrm{l}\right\}, $$

with n the number of Cartesian readouts per pseudo-spiral (φ_0_-φ_n_) and l the number of turns. In all experiments n =100 and l =3 were used. After one pseudo-spiral arm was sampled a new arm was acquired in a tiny golden-angle increment of 23.63 degrees relative to the previous pseudo-spiral. The number of pseudo-spirals depends on the desired acceleration factor. For asymmetric matrix size the pseudo-spiral was rescaled according to the ratio k_y,max_/k_z,max_.

A text file containing all (k_y_,k_z_)-profiles was created in MATLAB (MathWorks Inc., Natick, Massachusetts, USA) and imported in the scanner software (Philips Healthcare, Best, the Netherlands; software release 5.1.8 CDAS). A custom patch was used (PROspective Undersampling in multiple Dimensions (PROUD) patch [24–26]), which allows sampling of predefined trajectories. PROUD also enables continously updating the (k_y_,k_z_)-profiles within each cardiac cycle. Moreover, PROUD controls the acquisition in real time by bitmasking the k-t-space filling and prevents duplicate acquisitions of profiles of the same cardiac frame.

In continuous pseudo-spiral sampling, the acceleration factor R can be chosen in reference to the number of readouts of a fully sampled scan N_fully_ and the number of readouts of an undersampled scan N_undersampled_. Hereby N_fully_ depends on the number of phase encoding steps ${\mathrm{N}}_{k_y}$ and ${\mathrm{N}}_{k_z}$, the desired number of cardiac frames N_card_ and the number of flow encoding segments N_flow_.

2 $$ \mathrm{R}=\frac{{\mathrm{N}}_{\mathrm{fully}}}{{\mathrm{N}}_{\mathrm{undersampled}}}=\frac{{\mathrm{N}}_{k_y}\ast {\mathrm{N}}_{k_z}\ast {\mathrm{N}}_{\mathrm{card}}\ast {\mathrm{N}}_{\mathrm{flow}}}{{\mathrm{N}}_{\mathrm{undersampled}}} $$

### Compressed sensing reconstruction

All image reconstruction steps, including phase-offset corrections, were done offline using MRecon and GTFlow (Gyrotools, Zurich, Switzerland). For each measured k-line, the time distance to the ECG R top was available from the raw data. This allowed binning of the data into a specific number of cardiac frames (N_card_) using an ‘absolute’ binning approach. The last cardiac frames at end-diastole receive less data due to R-R variabilities and were discarded during flow curve analysis (phantom: last 2 frames see Fig. 3b, in vivo: last 3 frames due to larger R-R variability). Data from each flow-encoding direction was binned independently. After cardiac binning, the undersampled data was reconstructed with a non-linear CS-PI (compressed sensing and parallel imaging) algorithm available from the Berkeley Advanced Reconstruction Toolbox (BART) [27] according to

3 $$ \hat{\mathbf{m}}\underset{\kern2.5em \mathbf{m}}{=\arg\ \min}\left\{{\left\Vert {\mathrm{F}}_{\mathrm{U}}\mathbf{m}-\mathbf{y}\right\Vert}_2+\uplambda\ \mathrm{TV}\mathbf{m}\right\}, $$

using a sparsifying TV operator performed along the time dimension. F_U_ denotes the undersampling Fourier operator, **y** the measured data, and **m** the reconstructed data. While the left term ensures data consistency, the right term enforces sparsity, regularized by parameter λ*.* Reconstructions were tested for different regularization parameters and number of iterations i. Heuristically, by reconstructing the data using different settings (ranging from 0.001< λ < 1, and 2 < i < 100) λ =0.01 and i = 10 were found to lead to the best match in peak flow values in comparison to the 2D flow reference scan, and were thus used for all reconstructions.

### Flow phantom experiments

**Fig. 2:**
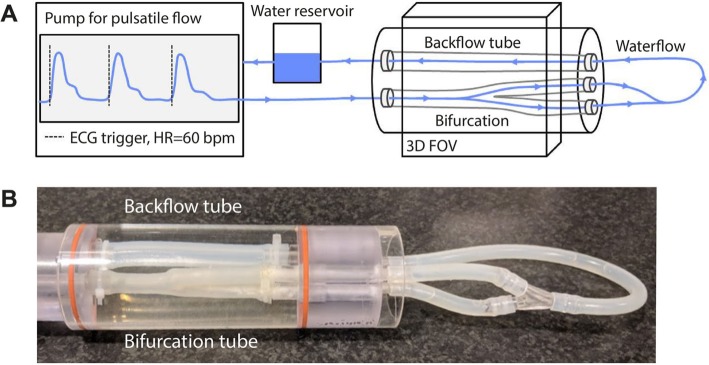
**a** The phantom setup. A pulsatile water flow enters the phantom in a compliant silicon tube in the shape of a carotid bifurcation and then returns in a straight silicon tube, which leads back to a reservoir. The water flow had a generated heart rate of 60 ± 5 bpm. The silicon tubes were embedded in water. **b** Image of the phantom tubes

A flow phantom was designed (LifeTec, Eindhoven, The Netherlands) (Fig. 2) consisting of a distensible silicon carotid artery embedded in water. A pulsatile water flow was applied at a simulated heart rate of 60 bpm and a random variability with 5% standard deviation. At the inlet of the phantom a peak flow rate of 10 ml/s and a mean flow rate of 3 ml/s was established.

4D flow CMR was performed with a 3 T CMR scanner (Ingenia, Philips Healthcare) using a 32-channel head coil and a referenced four-point flow encoding method (as explained in detail in [28]). Scan parameters were: TR = 8.9 ms, TE = 4.5 ms, FA = 8°, VENC = 150 cm/s (in all three flow encoding directions), matrix size = 160 × 160 × 40, FOV = 128 × 128 × 32 mm^3^, bandwidth = 713 Hz/pixel, spatial resolution = 0.8 × 0.8 × 0.8 mm^3^, and 19 reconstructed cardiac frames. No segmented k-space was used. As a flow reference, fully sampled 2D flow CMR scans with identical scan settings (except FA = 20° and bandwidth = 217 Hz/pixel) and spatial resolution = 0.8 × 0.8 × 3 mm^3^ were acquired in two planes perpendicular to the tubes. No contrast agent was used. The 4D flow scans were accelerated R = 2, 4, 6, 8, 10, 12, 15, and 20 times, with corresponding scan times decreasing from 45:57, 22:59, 15:18, 11:28, 9:08, 7:32, 5:45, to 4:30 min. The non-accelerated scan time for the given heart rate and number of cardiac frames amounted to 1:32 h.

### In vivo experiments

The acceleration technique was applied in the carotid arteries of 7 healthy subjects (27 ± 2 y, 4 male) using an 8-channel neck coil. Using the same settings as for the phantom, the 4D flow scans were accelerated R = 10, 20, 25 and 30 times (scan times: 9:08, 4:16, 3:14 and 2:32 min, respectively). Left and right carotid arteries were analyzed separately. 2D flow CMR reference scans were acquired using the same settings as for the phantom.

### Flow rate and WSS calculation

To calculate flow rates, regions-of interest (ROIs) in common, internal, and external carotid artery (CCA, ICA and ECA) were drawn using GTFlow in the same slice as the 2D flow reference scans for each artery and each acceleration factor separately. The time resolved flow rate Q was calculated as: Q(t) = ∫_ROI_v(**r**, t) d^2^**r**, with v(**r**, t) the absolute velocity at position **r** and cardiac phase t.

A 3D segmentation of the carotid bifurcation was created per 4D flow scan using Mimics (Materialise, Leuven, The Netherlands) for both phantom and in vivo scans. The peak systolic time frame was defined as the time frame with the highest absolute velocity averaged over the segmented volume. WSS vectors **τ**  were calculated in MATLAB (Mathworks) as described in Potters et al. [29]:

4 $$ \boldsymbol{\uptau} =2\upeta \dot{\upvarepsilon}\cdotp \mathbf{n}, $$

With η the blood viscosity of 3.2·10^− 3^ Pa·s, **n** the inward normal vector on the vessel wall and $\dot{\upvarepsilon\ }$ the rate of the deformation tensor

5 $$ \dot{\upvarepsilon} = \left[\ \begin{array}{ccc}\frac{\partial {\mathrm{v}}_{\mathrm{x}}}{\mathrm{\partial x}}& \frac{1}{2}\left(\frac{\partial {\mathrm{v}}_{\mathrm{y}}}{\mathrm{\partial x}}+\frac{\partial {\mathrm{v}}_{\mathrm{x}}}{\mathrm{\partial y}}\right)& \frac{1}{2}\left(\frac{\partial {\mathrm{v}}_{\mathrm{z}}}{\mathrm{\partial x}}+\frac{\partial {\mathrm{v}}_{\mathrm{x}}}{\mathrm{\partial z}}\right)\\ {}\frac{1}{2}\left(\frac{\partial {\mathrm{v}}_{\mathrm{x}}}{\mathrm{\partial y}}+\frac{\partial {\mathrm{v}}_{\mathrm{y}}}{\mathrm{\partial x}}\right)& \frac{\partial {\mathrm{v}}_{\mathrm{x}}}{\mathrm{\partial x}}& \frac{1}{2}\left(\frac{\partial {\mathrm{v}}_{\mathrm{z}}}{\mathrm{\partial y}}+\frac{\partial {\mathrm{v}}_{\mathrm{y}}}{\mathrm{\partial z}}\right)\\ {}\frac{1}{2}\left(\frac{\partial {\mathrm{v}}_{\mathrm{x}}}{\mathrm{\partial z}}+\frac{\partial {\mathrm{v}}_{\mathrm{z}}}{\mathrm{\partial x}}\right)& \frac{1}{2}\left(\frac{\partial {\mathrm{v}}_{\mathrm{y}}}{\mathrm{\partial z}}+\frac{\partial {\mathrm{v}}_{\mathrm{z}}}{\mathrm{\partial y}}\right)& \frac{\partial {\mathrm{v}}_{\mathrm{x}}}{\mathrm{\partial x}}\end{array}\right] $$

By selecting a local coordinate system for each point on the vessel wall such that the z′ axis aligns with the inward normal, and by assuming no flow through the vessel wall, i.e. **n** · **v** = 0, this equation simplifies to

6 $$ \boldsymbol{\uptau} =\upeta\ \left[\frac{\partial {\mathrm{v}}_{\mathrm{x}\prime }}{\mathrm{\partial z}^{\prime }}\kern0.75em \frac{\partial {\mathrm{v}}_{\mathrm{y}\prime }}{\mathrm{\partial z}^{\prime }}\kern0.5em 0\right], $$

Spatial derivatives of the velocity on the vessel wall were derived from fitted splines. After transforming back to the original coordinate system, the (time resolved) magnitude of the WSS vector is reported throughout the manuscript. The segmented volume of each scan was rigidly registered on the segmented carotid of the scan with the lowest acceleration factor (MATLAB) [30]. For in vivo scans this was done per subject. WSS and velocity values at peak systole were interpolated on the reference volume, to facilitate voxel-wise comparisons. Pathlines were visualized using GTFlow.

### Statistical analysis

Bland-Altman analysis was used to determine the mean difference and the limits of agreement. Orthogonal regression was used to determine the slope, intercept and Pearson correlation coefficient ρ.

## Results

### Phantom

**Fig. 3:**
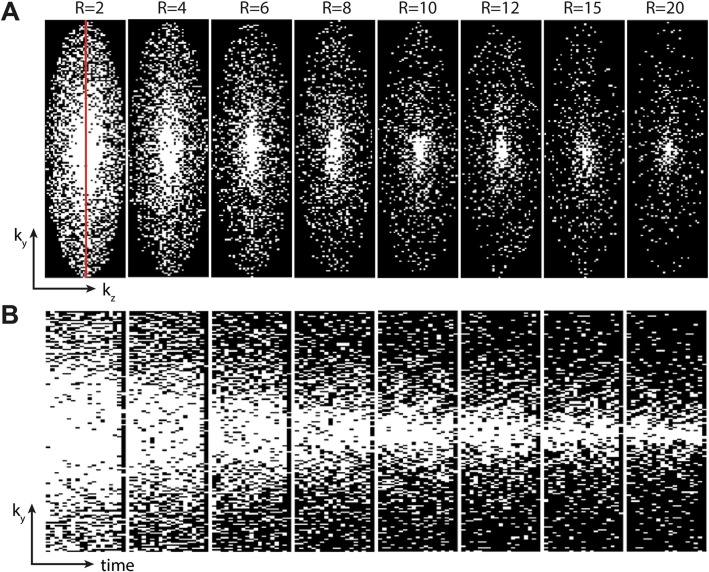
**a** Undersampling patterns in both phase encoding directions (k_y_,k_z_) for acceleration factors R = 2–20 as a result of cardiac binning. **b** Undersampling patterns for one phase encoding direction (k_y_) and 19 cardiac frames (time) for acceleration factors R = 2–20

Figure 3 shows typical k-t-space filling patterns in spatial and temporal direction with from left to right increasing acceleration factors of R = 2–20. For all the accelerations the resulting k-spaces after cardiac binning had quasi-random filling with a densely sampled center. The undersampling patterns were also incoherent over time (Fig. 3b), which is important for the CS reconstruction with TV constraint.

**Fig. 4:**
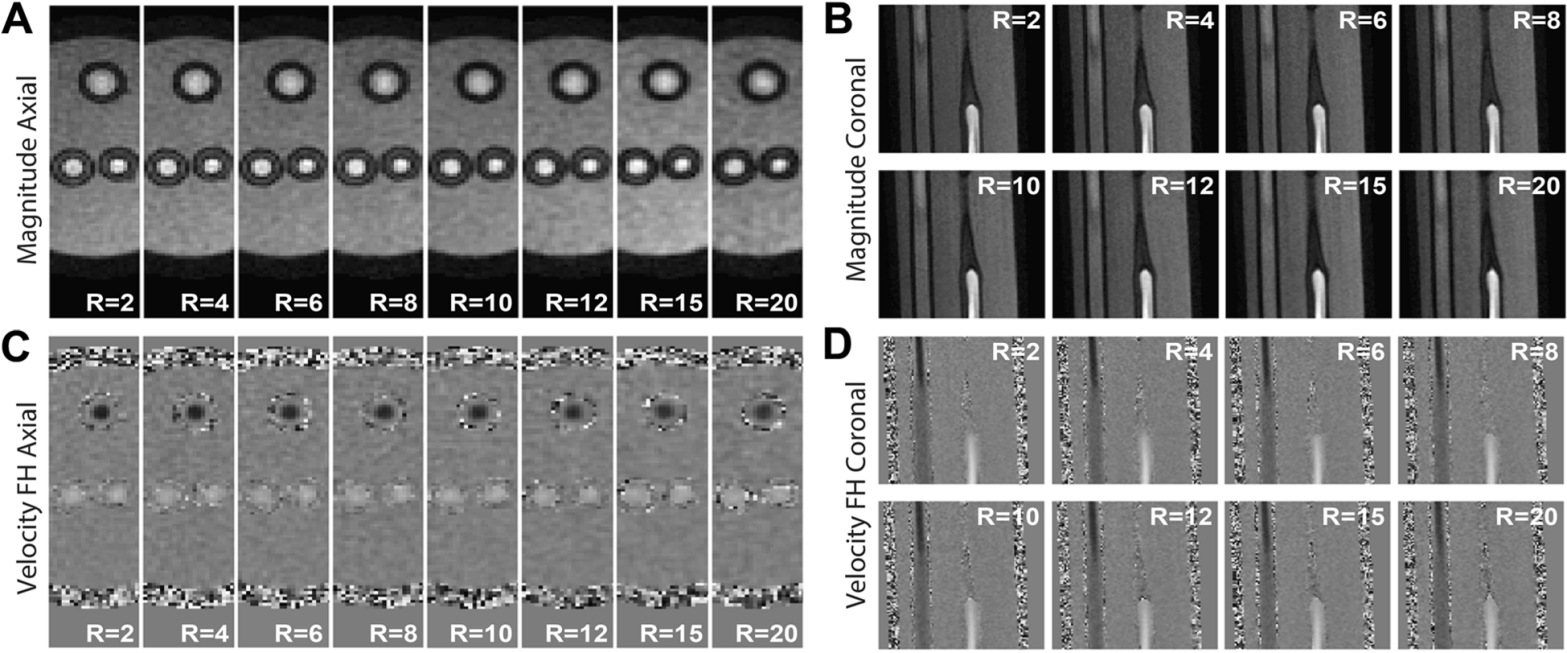
Axial (**a**) and coronal (**b**) view of magnitude images of the phantom for R = 2–20. Axial (c) and coronal (d) view of velocity images for R = 2–20. Both velocity images show feet-head (FH) flow encoding with a velocity range of − 150 to 150 cm/s. In axial images the backflow tube is on the top and the bifurcation tube on the bottom. In coronal images, the backflow is on the left and the bifurcation tube on the right

**Fig. 5:**
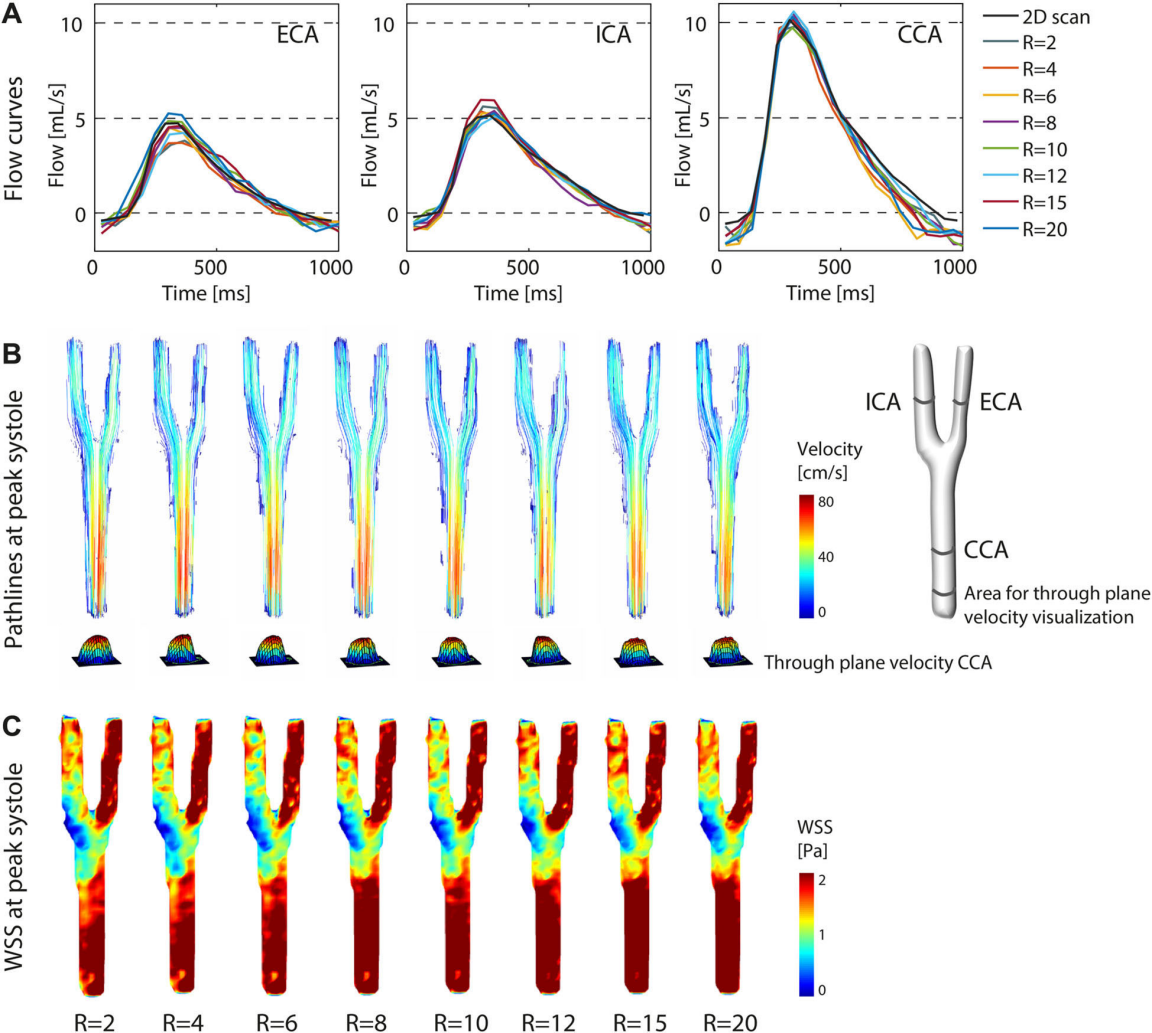
Flow in the carotid phantom at (**a**) external carotid artery (ECA), internal carotid artery (ICA) and common carotid artery (CCA) for acceleration factors R = 2–20 and the fully sampled 2D flow CMR scans. **b** Pathlines and wall shear stress (WSS) (**c**) in the carotid phantom for acceleration factors R = 2–20 during peak systole

Reconstructed magnitude and feet-head (FH) velocity images of the phantom visually were very comparable for all acceleration factors (Fig. 4a-d). With increasing acceleration factor some spatial blurring became apparent, particularly for the tube walls. More importantly however, the flow curves for ROIs in CCA, ICA and ECA were comparable for all acceleration factors and in comparison to the fully sampled 2D flow CMR scan (Fig. 5a). More variations in peak flow rate were observed in the smaller vessels (ICA and ECA), than in the CCA.

The largest deviation in flow rate as compared to the 2D reference was observed at peak systole in the CCA, with 0.5 ml/s (4.4%) less for the R = 12 times accelerated 4D flow CMR scan (Additional file 7: Table S1). The R = 20 times accelerated scan had a deviation in peak systolic flow rate of only 0.2 ml/s (1.7%). Pathlines, through plane velocity and WSS of the phantom at peak systole are shown in Fig. 5b, c (pathline movies are available online, see Additional file 1: Video S1). Only minor visual differences were observed for WSS values and pathlines at peak systole for scans with varying acceleration factors.

**Fig. 6:**
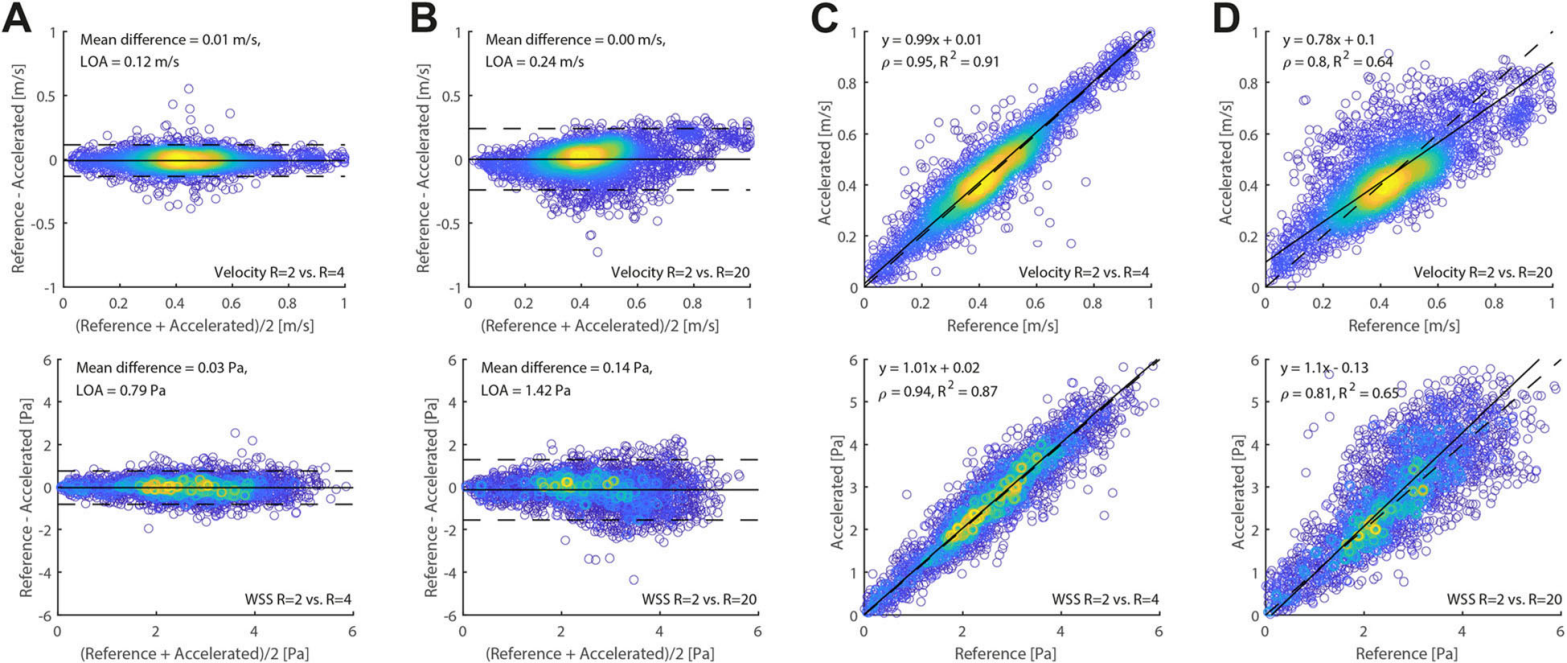
Bland-Altman plots and orthogonal regression for peak systolic WSS (top row) and velocity (bottom row) values of the phantom scan. The scan with the lowest acceleration factor (R = 2) is compared to acceleration factor R = 4 (**a** and **c**) and to acceleration factor R = 20 (**b** and **d**), to vizualize the changes occuring at higher undersampling rates

For a quantitative assessment of the influence of high accelerations on the hemodynamic parameters at peak systolic, velocity and WSS values in the phantom and per acceleration factor (R = 4–20) were compared voxel-wise within the segmented volume against the values of the lowest acceleration factor (R = 2) in a Bland-Altman analysis (Fig. 6).

**Fig. 7:**
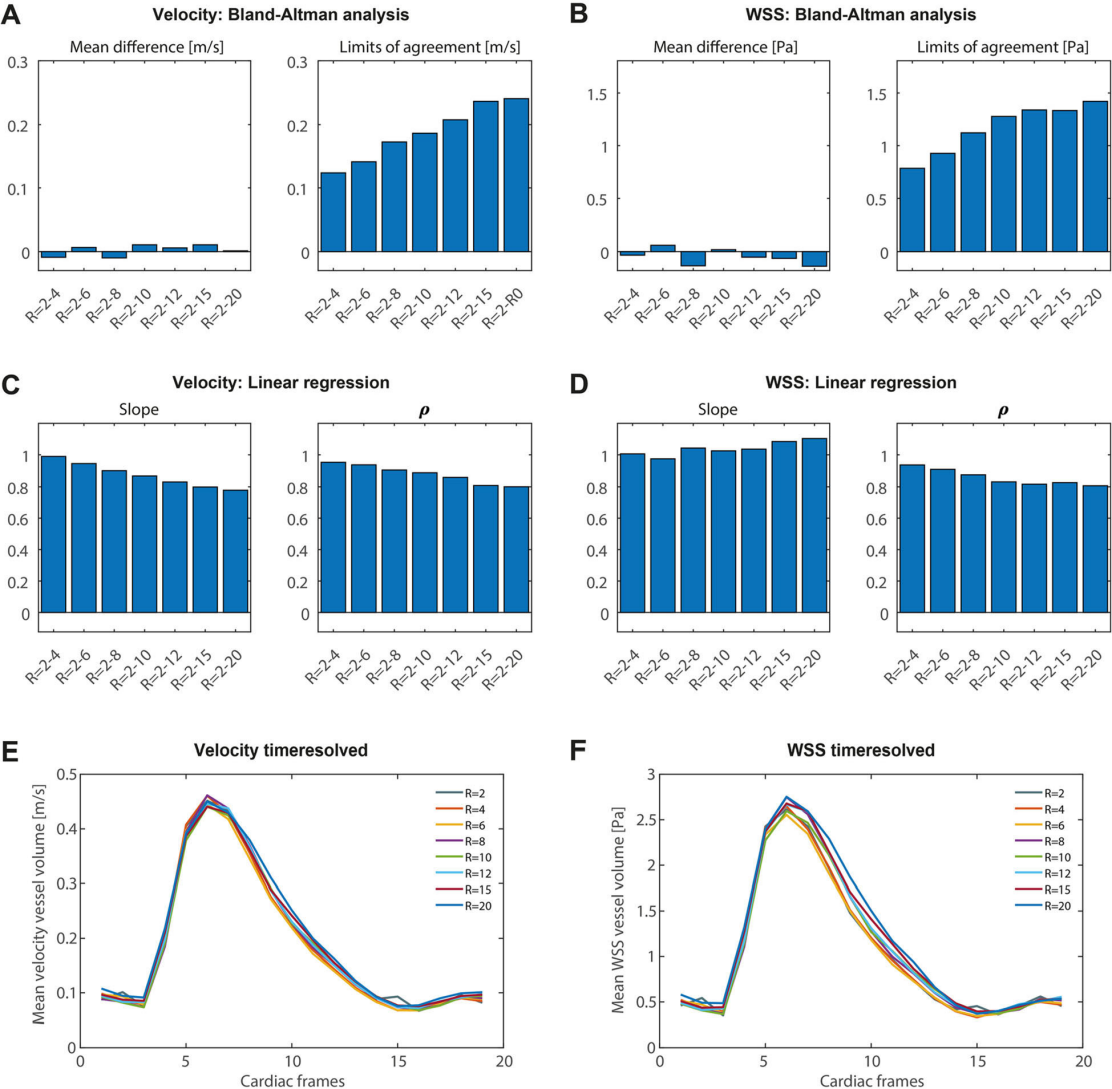
**a** Comparison of peak systolic WSS values at R = 2 with all higher acceleration factors R = 4–20 using Bland-Altman analysis (mean differences and limits of agreement (LOA)). **b** The same comparison as in (**a**) for velocities at peak systole. **c** Comparison of peak systolic WSS values at R = 2 with all higher acceleration factors R = 4–20 using orthogonal regression (slope of the regression line and Pearson correlation coefficient ρ). **d** The same comparison as in (**c**) for velocities at peak systole. **e-f** Mean WSS and mean velocity (calculated per time point and over the vessel volume), plotted for all cardiac frames

The mean differences and the limits of agreement (LOA) of the Bland-Altman analysis for all acceleration factors, shown in Fig. 6, are summarized and visualized in Fig. 7. The maximum mean difference for WSS at peak systole was 5.3% for R = 20. The maximum mean difference for velocity was − 2.4% for R = 10. The LOA increased slightly with increasing acceleration factor. Slope and Pearson correlation coefficients are summarized in Fig. 7c, d. The slope of the correlation for both WSS and velocity at peak systole essentially stayed the same with increasing acceleration, whereas the Pearson correlation coefficients ρ slightly decreased for higher acceleration factors. Figure 7e, f depicts the mean WSS and mean velocity of the vessel volume not only at peak systole, but for every time point in the cardiac cycle, demonstrating no variation in other cardiac frames. A summarizing table of the Bland-Altman analyses for peak systolic WSS and velocity values of the phantom experiment is found in Additional file 7: Table S2.

### In vivo

**Fig. 8:**
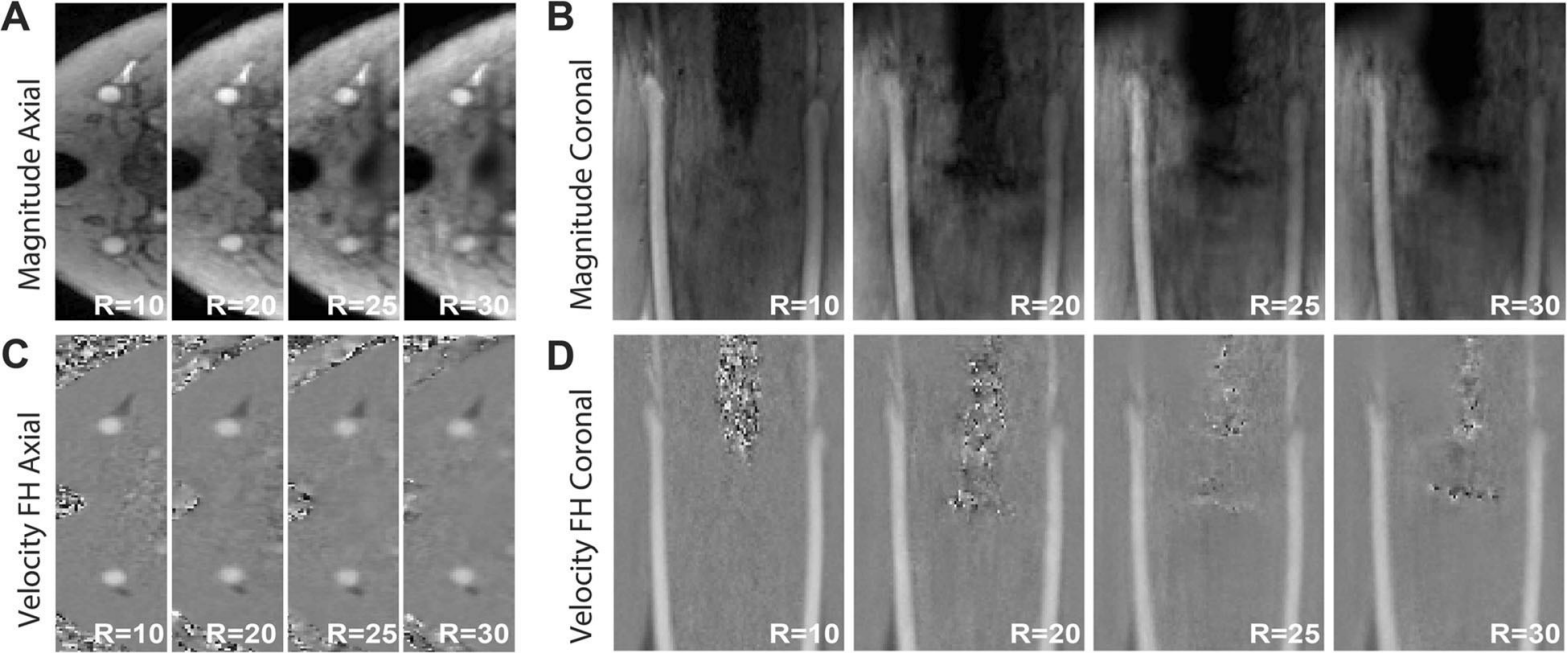
Axial (**a**) and coronal (**b**) views of one representative in vivo scan at acceleration factors R = 10–30. For both views velocity images in the range of − 150 to 150 cm/s are shown (**c**, **d**)

Similar to the phantom scans, the magnitude and velocity images of the in vivo scans were visually of good quality up to the highest acceleration factors (Fig. 8). Some blurring occurred with increasing acceleration.

**Fig. 9:**
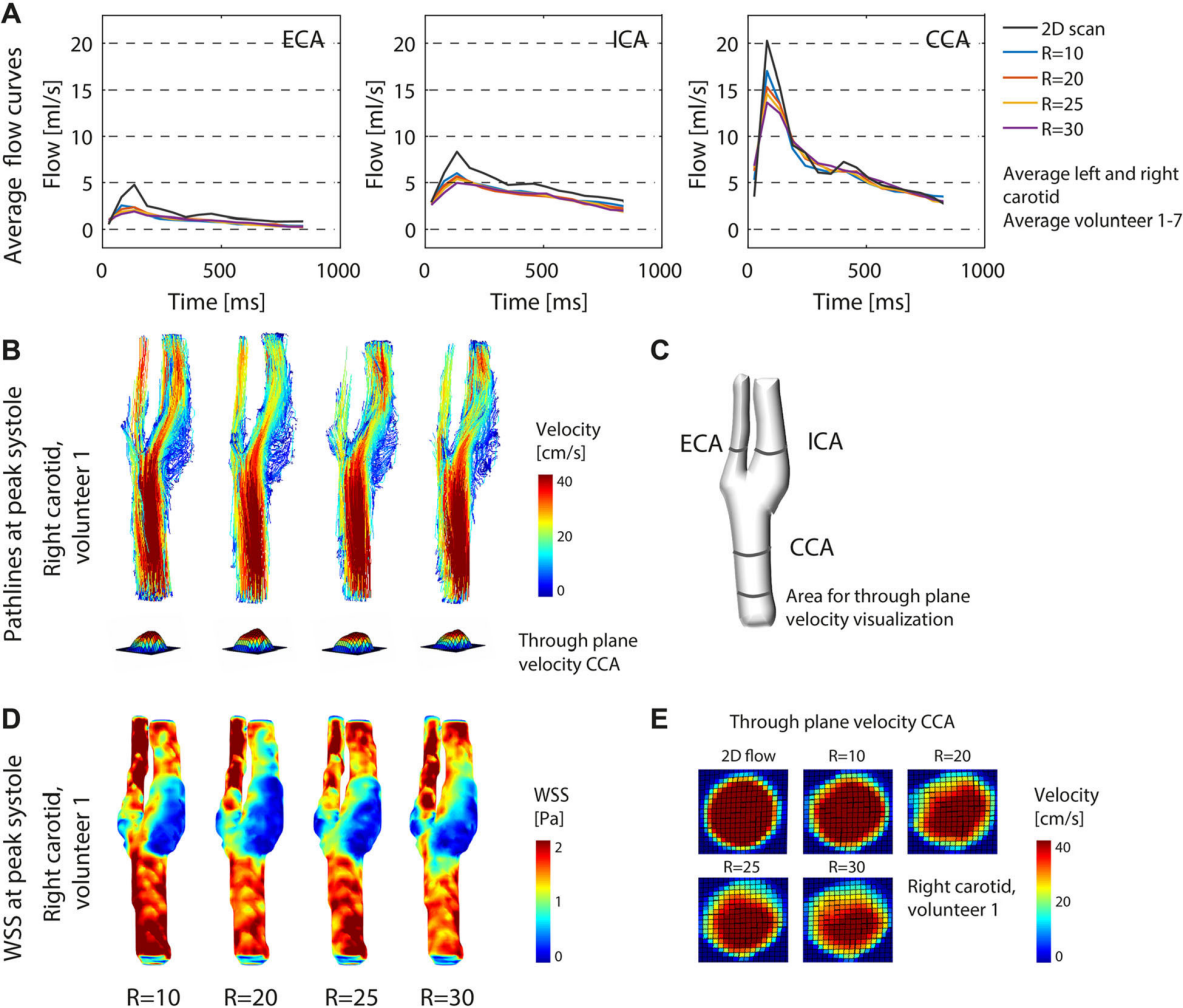
Flow curves in the (**a**) ECA, ICA and CCA, averaged for left and right carotid, plotted for all acceleration factors. The flow curves are averaged over all 7 subjects and are compared to a fully sampled 2D flow CMR scan at the same position (also averaged over all subjects). **b** Pathlines for all acceleration factors in the right carotid artery of one exemplary subject during peak systole. **c** Regions of interest (ROIs) used in (**a**). **d** WSS for all acceleration factors in the right carotid of one exemplary subject during peak systole. **e** Through plane velocities in the right CCA of one one exemplary subject during peak systole

Flow rate was calculated for the accelerated 4D flow CMR scans and compared to the fully sampled 2D flow CMR scan. The flow curves through the cardiac cycle, averaged over all subjects, and left and right carotid, plotted per acceleration factor, are shown in Fig. 9a. The typical carotid flow curves through the cardiac cycle could be appreciated for all the accelerated scans, with consistent flow rate values, albeit with lower flow rate values than for the 2D reference scan. The subject-averaged flow rate at peak systole with respect to the 2D flow reference was lower by − 5.0 ± 2.1 ml/s (− 24.4 ± 10.4%) for R = 20 and − 6.6 ± 3.4 ml/s (− 32.6 ± 16.7%) for R = 30 in the CCA (Additional file 7: Table S3). Flow rates in ICA and ECA were lower in accelerated 4D flow scans than in the 2D flow scan.

**Fig. 10:**
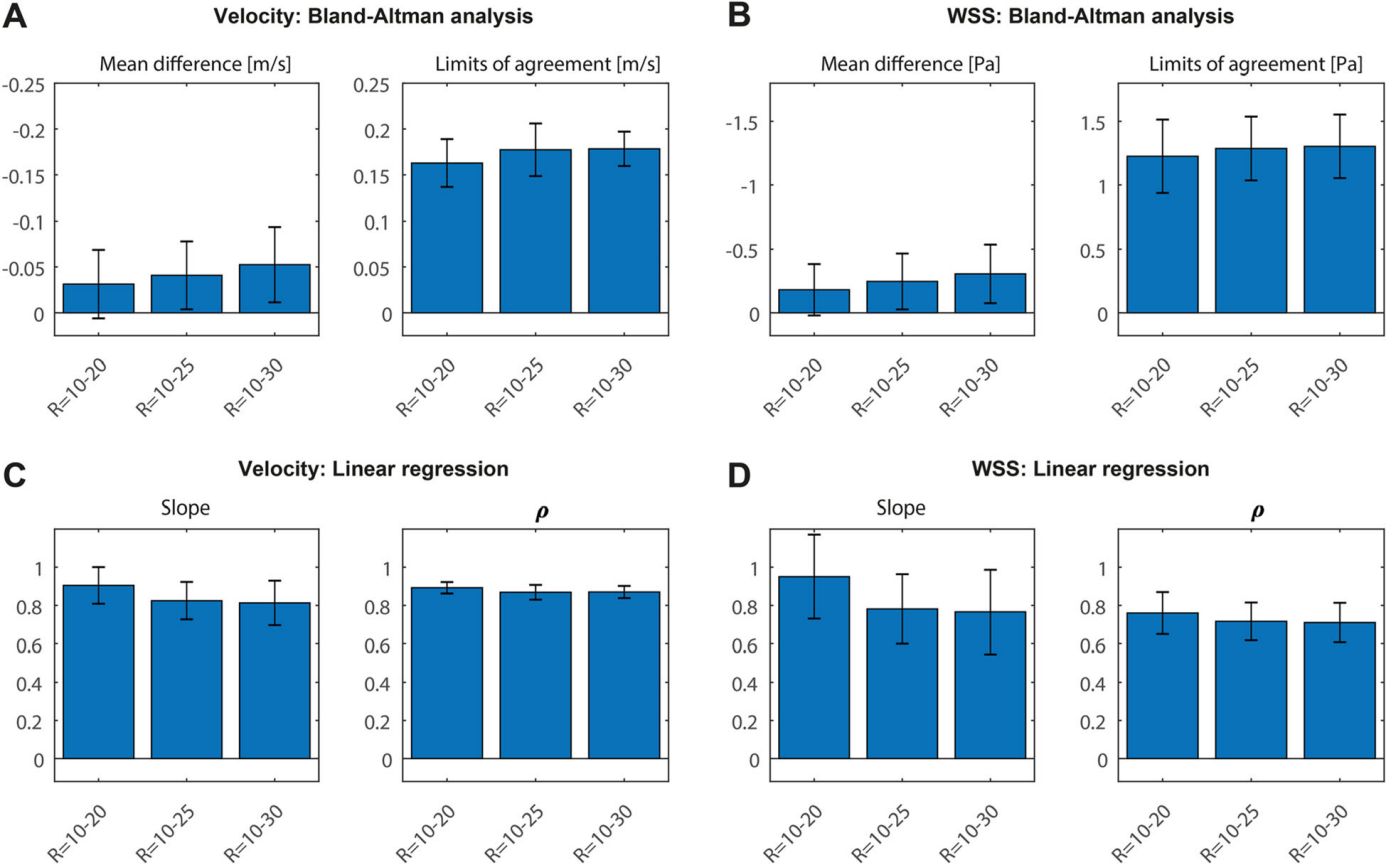
**a** Comparison of peak systolic WSS values at R = 10 with acceleration factors R = 20–30 using Bland-Altman analysis (mean differences and limits of agreement (LOA)). The averaged values of all subjects and both carotids are shown. The errorbars indicate the standard deviation between the different scans. **b** The same comparison as in (**a**) for velocities at peak systole. **c** Comparison of peak systolic WSS values at R = 10 with all higher acceleration factors R = 20–30 using orthogonal regression (slope of the regression line and Pearson correlation coefficient ρ). **d** The same comparison as in (**c**) for velocities at peak systole

Calculated pathlines, through plane velocities, and WSS during peak systole of one exemplary in vivo scan are shown in Fig. 9b-e. Pathlines movies during the cardiac cycle are also available online in Additional file 2: Video S2. Peak systolic WSS at all acceleration factors for all volunteers can be found in Additional file 3: Figure S3. Visually, peak systolic WSS spatial patterns, pathline movies, and through plane velocities were similar. However, in contrast to the phantom experiment, the mean WSS during peak systole from voxel-wise BA analysis decreased slightly for higher acceleration factors. WSS values decreased by − 9.9, − 13.4% and − 16.9% for acceleration factors of R = 20, 25 and 30, respectively (Fig. 10). Also, the slopes of the correlation plots and the Pearson correlation coefficients ρ slightly decreased for higher acceleration factors. The velocities within the vessel volume of the carotid arteries showed a similar trend. Velocities decreased by − 8.4, − 10.8%, and − 14.0% for acceleration factors of R = 20, 25 and 30, respectively (these results can also be found in Additional file 7: Table S4).

## Discussion

In this work we demonstrated the feasibility of accelerating 4D flow CMR of the carotids up to 30 times using a pseudo-spiral Cartesian sampling strategy. We recommend an acceleration factor of R = 20 to facilitate fast 4D flow CMR with an acceptable (< 10%) [31] underestimation of in vivo peak velocities (− 8.4 ± 9.9%) and WSS (− 9.9 ± 10.9%) in comparison to the R = 10 4D flow CMR reference scan. In comparison to 2D flow CMR, R = 20 times accelerated 4D flow CMR however may result in a peak flow rate underestimation of − 24.4 ± 10.4%. We developed and evaluated the protocol using a carotid flow phantom for acceleration factors up to R = 20 and in the carotid arteries of seven healthy subjects for acceleration factors up to R = 30. For high accelerations, we observed a trend of increased spatiotemporal blurring and decreased image quality in vivo. This resulted in a progressive decrease in the hemodynamic velocity and WSS values at systole.

Acceleration of 4D flow CMR has been an important topic since years. Experts in the field have published a consensus paper [32] in which different acceleration strategies for 4D flow CMR are advised. Papers from leading experts in accelerated 4D flow CMR [11, 12, 17, 33], as discussed one by one below, typically use a SENSE R = 2 acceleration (sometimes with a segmented k-space factor of 2–3) as a reference scan when validating a new acceleration technique. However, there exists no clear consensus on how to validate a 4D flow CMR acceleration techniques and which parameters to report in order to estimate accuracy [32]. Peak velocity or peak flow rate seem important parameters however peak velocity underestimation was not mentioned as an exclusion criterion in the consensus paper, as e.g. also k-space segmentation limits the temporal resolution of the scan, leading to peak underestimation. Nevertheless, peak velocity is important and should be considered when using acceleration. Generally, the accuracy of 4D flow CMR is also highly dependent on spatiotemporal resolution and SNR. There might also be a general bias between 4D and 2D flow CMR [32].

In Additional file 4: Figure S4A, we have repeated our experiments in vivo in *N* = 1 subject, including a SENSE (R = 2) 4D flow CMR acquisition with a k-space segmentation factor of 2. Our CS acceleration performs equally well to this reference method for acceleration factors up to R = 20. However both CS and SENSE accelerated 4D flow CMR scans underestimate peak flow rate values as compared to 2D flow CMR, which is likely associated with lower SNR (see also magnitude images in Additional file 4: Figure S4A, left). In a phantom setting (Additional file 4: Figure S4B) both methods perform evenly good up to an acceleration factor of R = 30. Phantom flow curves up to an acceleration factor of R = 100 are shown. This shows, that R = 10 CS accelerated scans serve as a good reference for scans with higher acceleration factors. It also demonstrates, that very high acceleration factors (> 50) can be achieved, in which reasonable flow curves can still be recognized.

A number of 4D flow CMR studies have reported on the accuracy of acceleration techniques. Cheng et al. demonstrated 4D flow in pediatric congenital heart disease patients, using a prospective pseudo-spiral sampling strategy with total variation CS reconstruction (VDRad). Effective acceleration factors ranged from R = 15–27. This resulted in clinically acceptable image quality, as rated by a radiologist. However, no quantitative comparisons with 2D flow were available. 4D flow CMR scans were compared against a CS reconstruction using spatial wavelets after (temporally constant) Poisson disk sampling [17], which had been demonstrated to lead to 12% flow rate underestimation in comparison to 2D flow at R = 5 in a previous study [34]. Valvano et al. prospectively accelerated 4D flow CMR in the aorta using a low-rank+sparse reconstruction algorithm. Errors in the aortic peak flow rate were reported in comparison to a standard SENSE = 2 acquisition (segmented k-space factor = 3). The peak flow rate was underestimated at an acceleration factor of R = 8 by 2.5 ± 4.6% in the ascending and by 3.6 ± 8.4% in the descending aorta [33]. Knobloch et al. [11] investigated the effects of k-t PCA acceleration on 4D flow CMR in the carotids (spatial resolution 1.2 × 1.2 × 1.2 mm^3^ vs. 0.8 × 0.8 × 0.8 mm^3^ in our study). Errors in peak flow rate were smaller than 4.9 ± 7%. However, the accelerated scans resulted from retrospective undersampling of a 4D flow CMR reference (75% Fourier sampling in k_y_ and k_z_, 23 min scan time), which likely will result in a better agreement between accelerated and reference scans than would be obtained for true prospective acceleration [11]. In Giese et al., prospectively k-t PCA accelerated aortic 4D flow CMR was compared to 2D flow [12]. The authors introduced an error metric E_SV_ for the stroke volume (SV) to compare 4D flow with 2D flow CMR (eq. 2 from [12]) and found E_SV_ = 2.5 ± 8.4 ml (5.6 ± 14.9%) for R = 8 in the aorta. Applying this metric to our in vivo carotid artery measurements, we find E_SV_ = 0.2 ± 0.7 ml (1.8 ± 10.9%) for R = 10, E_SV_ = 0.3 ± 0.5 ml (4.6 ± 7.0%) for R = 20, E_SV_ = 0.4 ± 0.4 ml (5.7 ± 6.7%) for R = 25 and E_SV_ = 0.4 ± 0.5 ml (5.6 ± 7.8%) for R = 30 in comparison to 2D flow. This time-averaged error metric can therefore partially obscure peak velocity differences. Taken together, the above studies show that there is no consensus on how to assess flow errors for accelerated 4D flow CMR protocols. The numbers will depend on whether the scans were retrospectively or prospectively undersampled and the choice of reference scan, being another accelerated 4D flow technique or 2D flow CMR.

In our study we found an underestimation of the in vivo carotid peak systolic flow rate for high acceleration factors in the CCA (− 24.4% for R = 20 and − 32.6% for R = 30) when compared to a 2D phase-contrast reference scan. This underestimation was less in phantoms, in which we found only − 1.7% peak flow rate underestimation (R = 20) in comparison to the 2D flow scan. The difference between in vivo and phantom scans might be explained by smoother flow profiles in the phantom, which might lead to better results when using a total variation operator in the CS reconstruction algorithm. The phantom curves, although very consistent between scans, showed slight variations due to partial volume effects with the tube wall, which had a noisy velocity due to lack of signal (Fig. 4). Differences between 2D flow and 4D flow CMR might be affected by higher SNR in 2D flow scans. Also, the high contrast of blood vessels in the 2D flow scan, created by inflow enhancement at high flip angles, might result in different partial volume effects and phase offsets than in 4D flow CMR [31, 35]. This might also explain deviations in smaller vessels like the ICA and ECA. Within the set of accelerated 4D flow CMR scans, volumetric velocity differences between R = 10 and higher accelerated scans were much more acceptable (8.4% for R = 20 and 14.0% for R = 30) than the results from the flow analysis. For peak systolic WSS, a similar behavior was observed. As the overall trend was to underestimate WSS at very high acceleration factors (similar to velocity), this could conceal small changes in WSS patterns that might be important for disease characterization. Overall we can conclude that, although hemodynamic parameters seem systematically underestimated if we consider the 2D phase contrast scan as a gold standard, the accuracy of WSS and velocity values with decreasing scan time in 4D flow is maintained within acceptable limits for R = 20. This can also be appreciated in the pathline movies, which remain visually of good quality up to the highest acceleration. Therefore, also clinical 4D flow CMR scans with acceleration factors of up to R = 20 can be used. However, depending on the purpose of the clinical examination, lower (for accurate peak velocity estimation) or higher (for mean flow, stroke volume or streamline visualization) acceleration factors could be considered. Moreover, regional differences in hemodynamic parameters are consistent for all accelerations, as for example shown in Fig. 9d (e.g. low WSS in the carotid bulb vs. high WSS in the rest of the vessel). More advanced pulsatile flow phantoms of the (diseased) carotid artery, with a sharp, systolic peak flow will help to investigate on the effects of 4D flow CMR acceleration in carotid pathology in the future.

Using a repeated scan protocol in vivo (repeated 2 times for R = 10, 20, 25 and 30) and in the phantom (repeated 3 times for R = 10, 15, 20, 25 and 30) as summarized in Additional file 5: Figure S5 we could demonstrate, that there was almost no deviation between repeated scans in both cases. The SD of paired differences (scan-rescan) per time frame was in vivo SD = 0.6, 0.6, 0.6, 0.6 ml/s and in the phantom SD = 0.4, 0.4, 0.4, 0.5 and 0.6 ml/s, for each acceleration factor respectively.

The comparison between in vivo and phantom experiments has some limitations. Although flow rate and mean velocities were similar, maximum velocities of 120 cm/s were achieved in the phantom in comparison to maximum velocities of 80 cm/s in vivo (see Additional file 6: Figure S6B-C). Because excessively long scan times are an issue in vivo, it was not possible to acquire 4D flow CMR data with low or no acceleration and consequently an in vivo 4D flow CMR dataset of for example R = 2 was not available. Another limitation to the study is that only a low number of *N* = 7 participants were included.

The here reported acceleration factors are calculated before the data acquisition in reference to the scan time of a fully sampled scan acquired with the same acquisition strategy. Effective acceleration factors can be calculated retrospectively to the scan from the ratio of the number of k-lines in a fully sampled k-space versus the actually sampled k-lines, i.e. N_undersampled_ in eq. [2] can vary slightly retrospectively to the scan. For in vivo scans R_effective_ (±SD) was on average R = 12.2 ± 0.8, R = 22.6 ± 0.9, R = 27.9 ± 0.9, and R = 32.9 ± 0.9, for scans with target acceleration R = 10, R = 20, R = 25, and R = 30, respectively. The effective acceleration factor would also vary slightly with the heart rate of each subject. This behavior is however similar in fully sampled scans, which are only optimal when the number of reconstructed cardiac frames times 4xTR (4 flow encoding steps used) approximately matches the average acquired heart cycle length. If the number of reconstructed cardiac frames is higher, undersampling would be the consequence, which in practice is compensated for by a retrospective interpolation. If the number of reconstructed cardiac frames is lower, oversampling occurs, which results in averaging and higher SNR, but which also leads to a lower temporal resolution. Reporting effective acceleration factors and their variations is similar to variations in regular cardiac triggered scans and therefore not further discussed in this paper.

Potential other applications for the here presented methodology are cine CMR scans and 4D flow CMR scans in other anatomical regions, such as the circle of Willis, the heart, the aorta and for assessing venous flow return. Other, cutting edge algorithms, such as low-rank [33] reconstruction, could be integrated in the current reconstruction to push the acceleration factors even further or mitigate the spatiotemporal blurring. The pseudo-spiral sampling strategy can be combined with breathing navigators or self-gating, which allows for sorting the data in respiratory bins [21, 36].

## Conclusion

We achieved highly accelerated 4D flow CMR of the carotid arteries using pseudo-spiral Cartesian undersampling and a CS reconstruction. At an acceleration factor of R = 20 the underestimation of peak velocity and peak WSS was acceptable (< 10%) in comparison to an R = 10 accelerated 4D flow CMR reference scan. Peak flow rates were underestimated in comparison with 2D flow CMR and decreased systematically with higher acceleration factors. However, even at an acceleration factor of R = 30, the highly CS accelerated 4D flow CMR scans allowed reconstructions of the 4D velocity field and velocity pathlines, at only 2:30 min scan time.

## Supplementary information

**Additional file 1: Video S1.** Pathline movies in the phantom carotid artery for all acceleration factors.

**Additional file 2: Video S2.** Pathline movies in the left carotid artery of an examplary healthy volunteer for all acceleration factors.

**Additional file 3: Figure S3.** WSS in the left and right carotid arteries of all seven in vivo experiments. An individual segmentation of the carotid artery was done per volunteer and acceleration factor.

**Additional file 4: Figure S4.** (**A**) Magnitude images, flow curves and throughplane velocities of an in vivo scan of *N* = 1 volunteer at CS acceleration factors R = 6 to R = 30, following the same scan protocol as described in the Methods section. Next to the CS acceleration a 2D reference scan and a 4D flow reference scan (SENSE = 2 and segmented k-space factor = 2) are shown. (**B**) Flow curves of a phantom scan for CS acceleration factors of R = 1 to R = 100. Next to the CS acceleration a 2D reference scan and a 4D flow reference scan (SENSE = 2 and segmented k-space factor = 2) are shown. Additionally, the peak flow rate as a function of the acceleration factor is shown.

**Additional file 5: Figure S5.** (**A**) Flow curves of an in vivo scan-rescan setting with 1 rescan for acceleration factors R = 10, 20, 25, 30. (**B**) Flow curves of a phantom scan-rescan setting with 2 rescans for acceleration factors R = 10, 15, 20, 25, 30.

**Additional file 6: Figure S6.** (**A**) Flow curves in the left and right carotid arteries of all seven in vivo experiments. (**B**) In vivo: Volunteer-averaged flow rate, through-plane velocity and maximum through-plane velocity for acceleration factors of R = 10–30. (**C**) Phantom: Flow rate, through-plane velocity and maximum through-plane velocity for acceleration factors of R = 2–20.

**Additional file 7: Table S1.** Flow rate differences of all accelerated scans in comparison to the 2D reference scan in the phantom experiment. **Table S2.** Statistical results from the Bland-Altman analysis and orthogonal regression for WSS and velocity in the phantom experiment. **Table S3.** Flow rate and velocity differences of all accelerated scans in comparison to the 2D reference scan as an average of all in vivo experiments. **Table S4.** Statistical results from the Bland-Altman analysis and orthogonal regression for WSS and velocity as an average of all in vivo experiments.

## Data Availability

All data was scanned using our in-house developed AMC ‘PROspective Undersampling in multiple Dimensions’ (PROUD) patch. A compiled version of this patch as well as all data and PWV analysis code are is available on reasonable request.

## Abbreviations

2D: Two dimensional
CCA: Common carotid artery
CMR: Cardiovascular magnetic resonance
CS: Compressed sensing
ECA: External carotid artery
ECG: Electrocardiography
FA: Flip angle
FH: Foot-head direction
FOV: Field of view
ICA: Internal carotid artery
LOA: Limits of agreement
PC: Phase contrast
PROUD: Scanner patch to PROspective Undersampling in multiple Dimensions
ROI: Region of interest
SD: Standard deviation
SNR: Signal to noise ratio
SV: Stroke volume
TE: Echo time
TR: Repetition time
TV: Total variation
VENC: Velocity encoding gradient
WSS: Wall shear stress
