# Supplementary figures and images for: Highly accelerated 4D flow cardiovascular magnetic resonance using a pseudo-spiral Cartesian acquisition and compressed sensing reconstruction for carotid flow and wall shear stress
Source: J Cardiovasc Magn Reson. 2020 Jan 20;22:7. doi: 10.1186/s12968-019-0582-z (PMC6971939; doi:10.1186/s12968-019-0582-z)

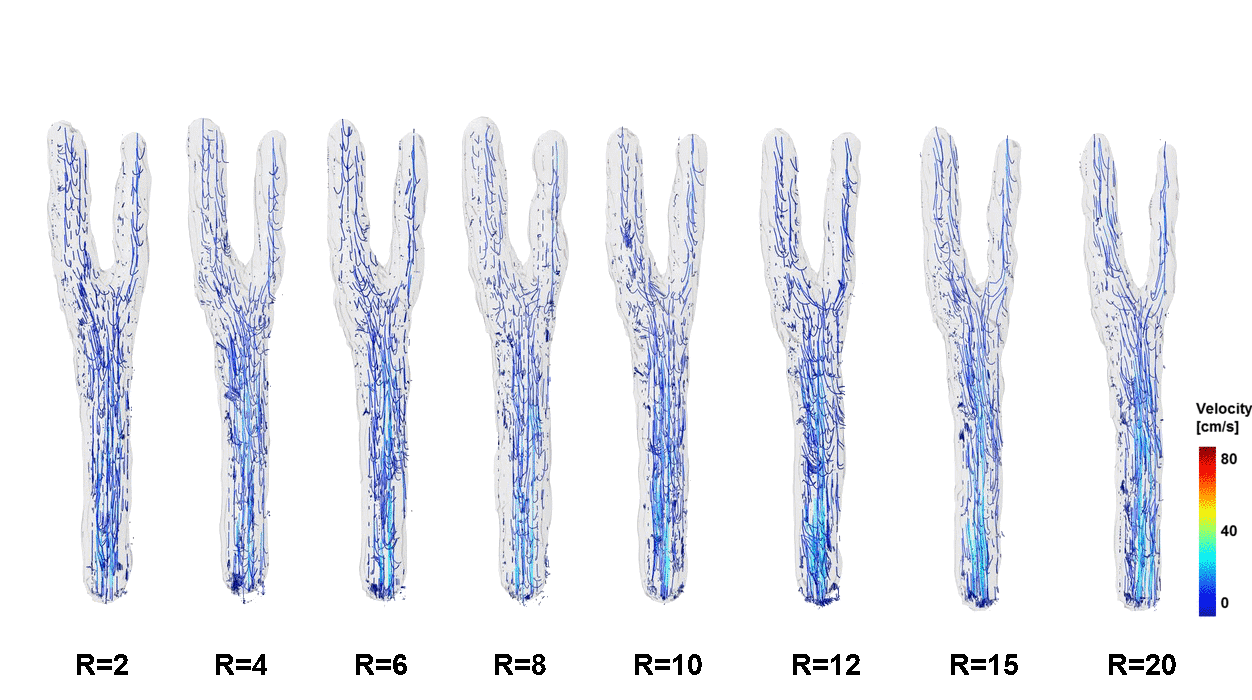

Supplement: Supplementary file 1 — Additional file 1: Video S1. Pathline movies in the phantom carotid artery for all acceleration factors. [file 12968_2019_582_MOESM1_ESM.gif]

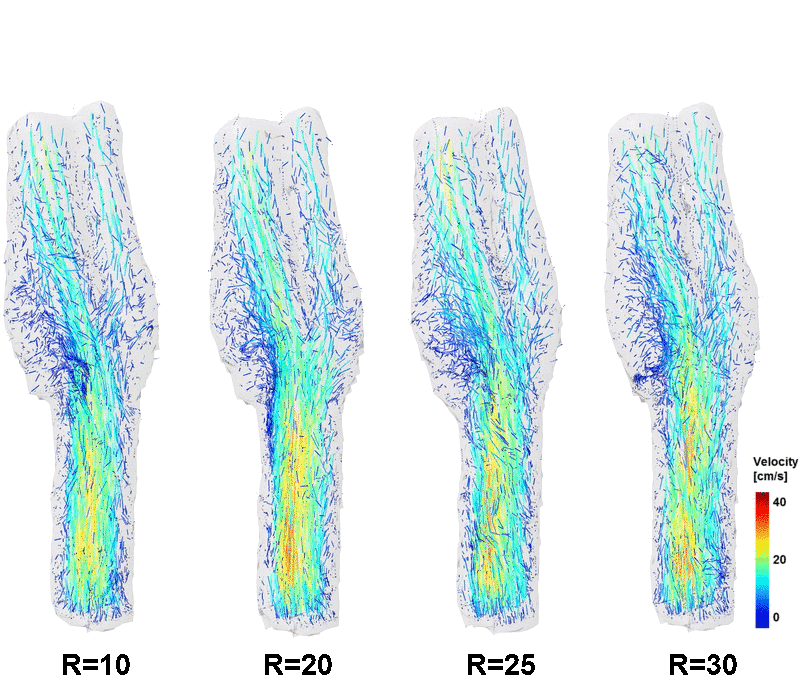

Supplement: Supplementary file 2 — Additional file 2: Video S2. Pathline movies in the left carotid artery of an examplary healthy volunteer for all acceleration factors. [file 12968_2019_582_MOESM2_ESM.gif]

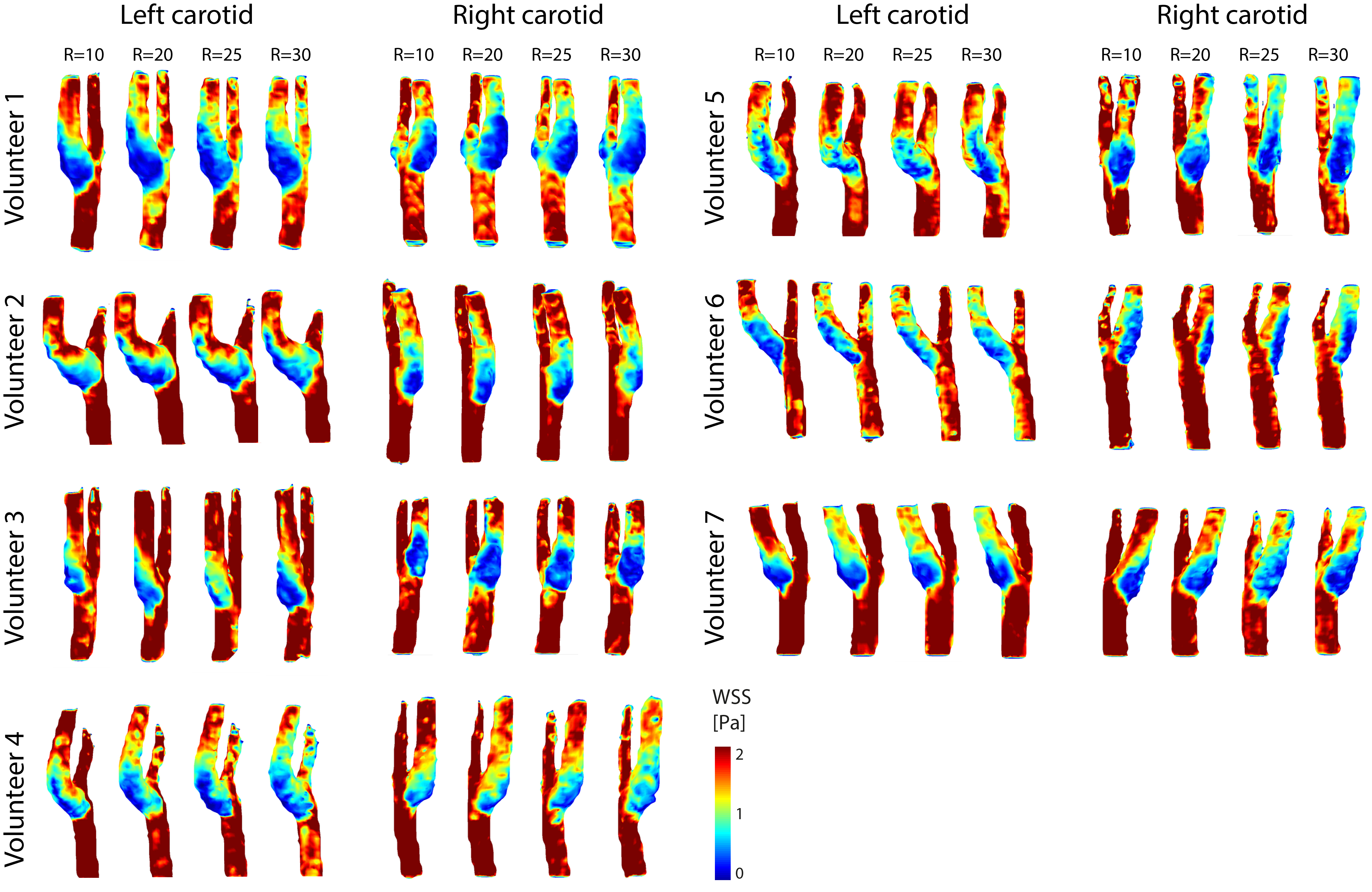

Supplement: Supplementary file 3 — Additional file 3: Figure S3. WSS in the left and right carotid arteries of all seven in vivo experiments. An individual segmentation of the carotid artery was done per volunteer and acceleration factor. [file 12968_2019_582_MOESM3_ESM.jpg]

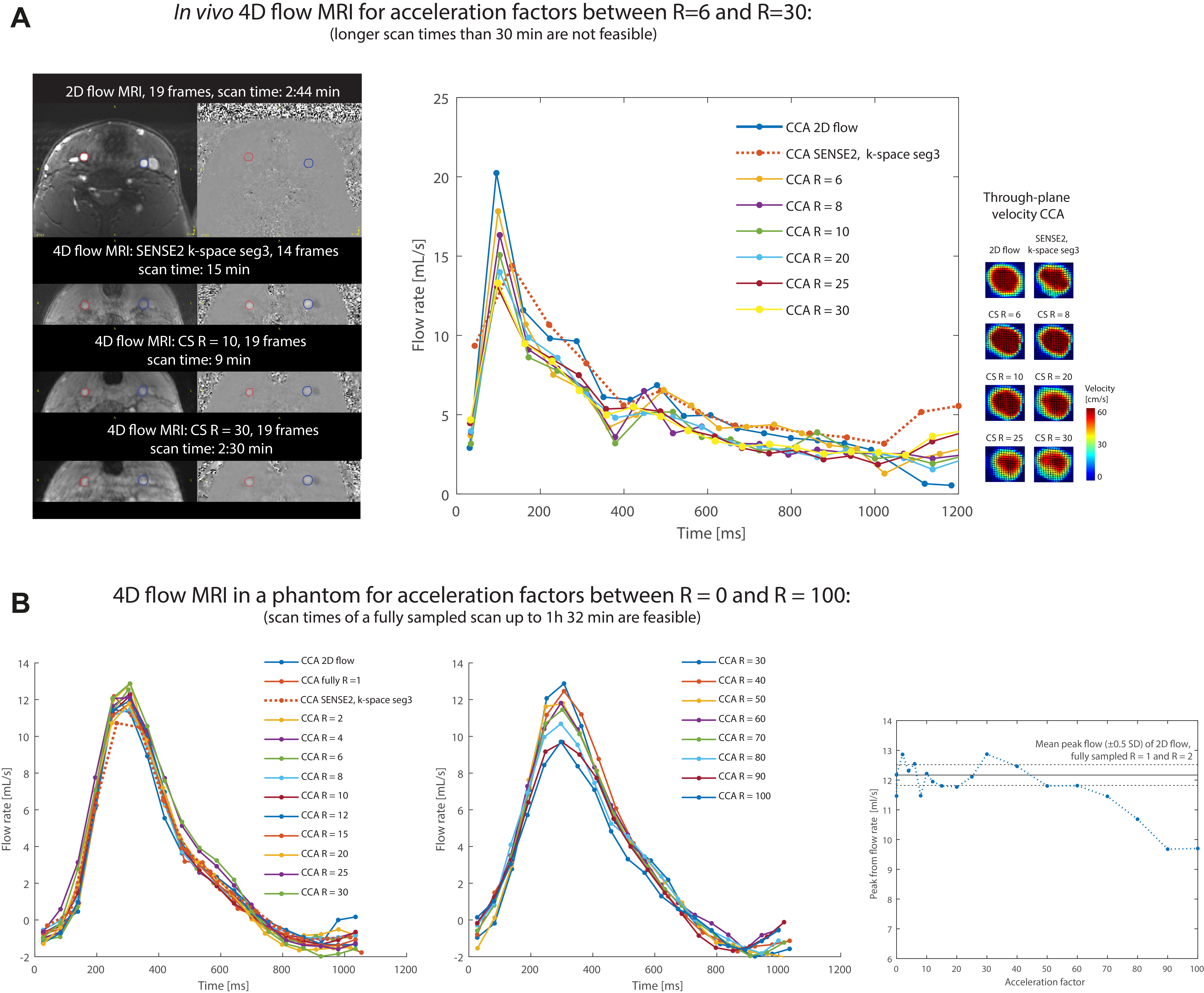

Supplement: Supplementary file 4 — Additional file 4: Figure S4. (A) Magnitude images, flow curves and throughplane velocities of an in vivo scan of N = 1 volunteer at CS acceleration factors R = 6 to R = 30, following the same scan protocol as described in the Methods section. Next to the CS acceleration a 2D reference scan and a 4D flow reference scan (SENSE = 2 and segmented k-space factor = 2) are shown. (B) Flow curves of a phantom scan for CS acceleration factors of R = 1 to R = 100. Next to the CS acceleration a 2D reference scan and a 4D flow reference scan (SENSE = 2 and segmented k-space factor = 2) are shown. Additionally, the peak flow rate as a function of the acceleration factor is shown. [file 12968_2019_582_MOESM4_ESM.jpg]

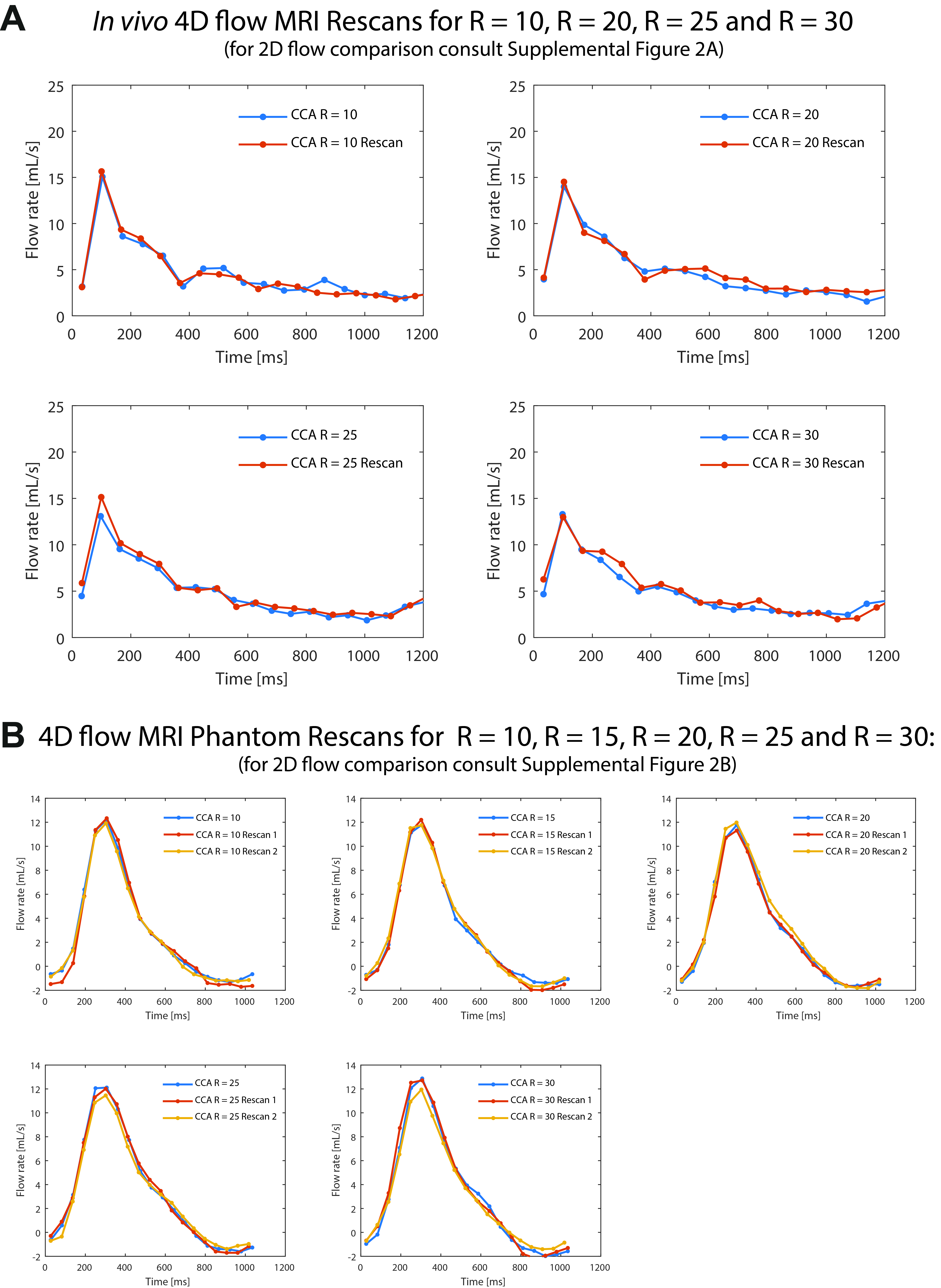

Supplement: Supplementary file 5 — Additional file 5: Figure S5. (A) Flow curves of an in vivo scan-rescan setting with 1 rescan for acceleration factors R = 10, 20, 25, 30. (B) Flow curves of a phantom scan-rescan setting with 2 rescans for acceleration factors R = 10, 15, 20, 25, 30. [file 12968_2019_582_MOESM5_ESM.jpg]

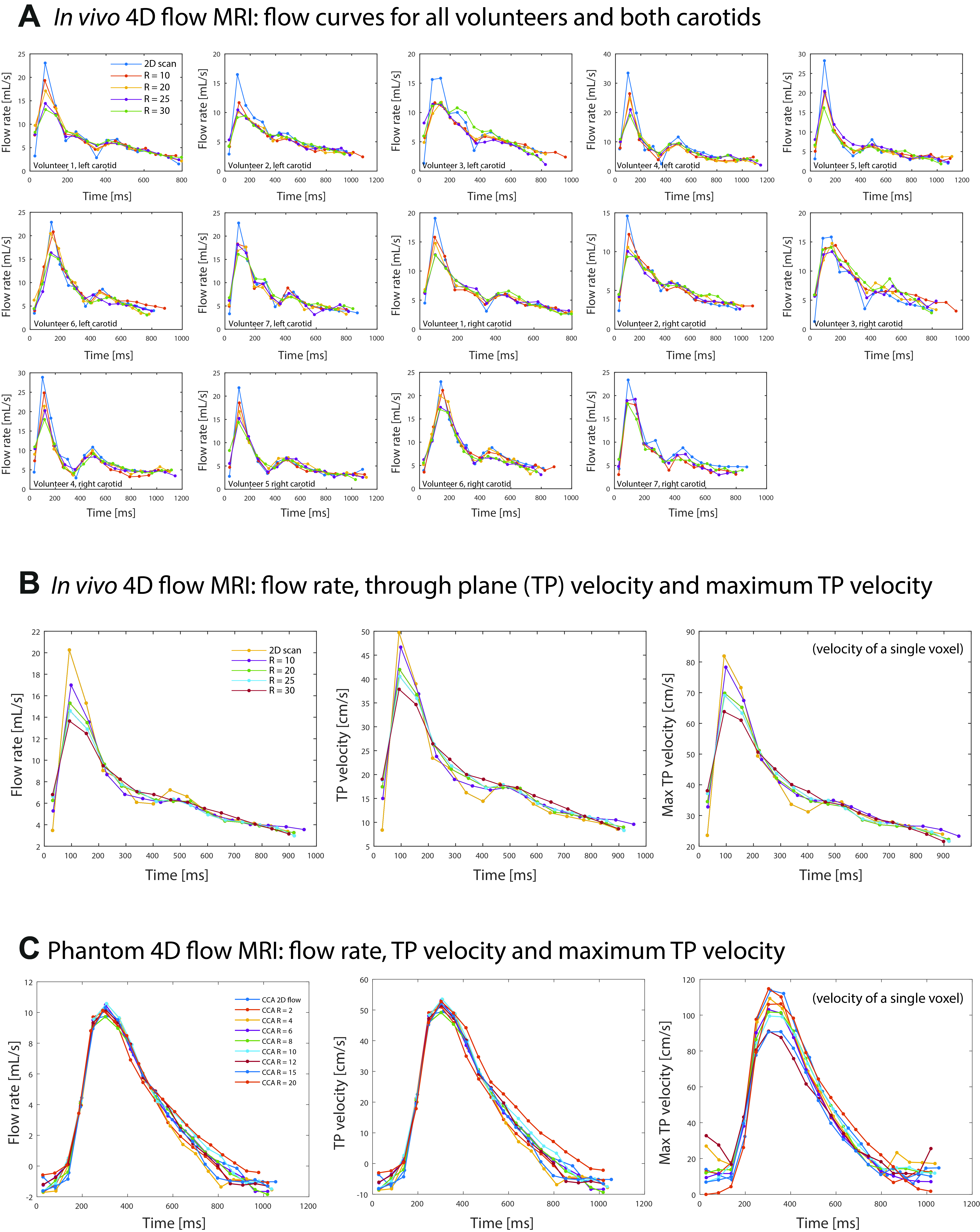

Supplement: Supplementary file 6 — Additional file 6: Figure S6. (A) Flow curves in the left and right carotid arteries of all seven in vivo experiments. (B) In vivo: Volunteer-averaged flow rate, through-plane velocity and maximum through-plane velocity for acceleration factors of R = 10–30. (C) Phantom: Flow rate, through-plane velocity and maximum through-plane velocity for acceleration factors of R = 2–20. [file 12968_2019_582_MOESM6_ESM.jpg]
