# Supplementary material for: Highly accelerated 4D flow cardiovascular magnetic resonance using a pseudo-spiral Cartesian acquisition and compressed sensing reconstruction for carotid flow and wall shear stress
Source: J Cardiovasc Magn Reson. 2020 Jan 20;22:7. doi: 10.1186/s12968-019-0582-z (PMC6971939; doi:10.1186/s12968-019-0582-z)
Supplement: Supplementary file 7 — Additional file 7: Table S1. Flow rate differences of all accelerated scans in comparison to the 2D reference scan in the phantom experiment. Table S2. Statistical results from the Bland-Altman analysis and orthogonal regression for WSS and velocity in the phantom experiment. Table S3. Flow rate and velocity differences of all accelerated scans in comparison to the 2D reference scan as an average of all in vivo experiments. Table S4. Statistical results from the Bland-Altman analysis and orthogonal regression for WSS and velocity as an average of all in vivo experiments. [file 12968_2019_582_MOESM7_ESM.docx]

**Table S1**

| **Phantom: Differences between**  **2D flow and R = 2/4/6/8/10/12/15/20** | |
| --- | --- |
| **Peak flow rate CCA:** | Difference  [ml/s] *(%*)* |
| 2D scan vs. R = 2 | -0.4 *(-3.4)* |
| 2D scan vs. R = 4 | -0.1 *(-0.6)* |
| 2D scan vs. R = 6 | 0.4 *( 4.3)* |
| 2D scan vs. R = 8 | 0.4 *( 3.6)* |
| 2D scan vs. R = 10 | -0.4 *(-4.1)* |
| 2D scan vs. R = 12 | 0.5 *( 4.4)* |
| 2D scan vs. R =15 | 0.1 *( 0.6)* |
| 2D scan vs. R = 20 | 0.2 *( 1.7)* |

* Relative to the 2D scan

**Table S2**

| **Phantom: Differences between R = 2 and R = 4/6/8/10/12/15/20** | | | | | | | |
| --- | --- | --- | --- | --- | --- | --- | --- |
|  | Bland-Altman | |  | Orthogonal regression | |  | Correlation |
| **WSS:** | Mean difference  [Pa] *(%*)* | LOA  [Pa] |  | Slope | Intercept  [Pa] |  | ρ |
| R = 2 vs. R = 4 | 0.03 *( 1.29)* | 0.79 |  | 1.01 | 0.02 |  | 0.94 |
| R = 2 vs. R = 6 | -0.06 *(-2.29)* | 0.93 |  | 0.97 | 0.01 |  | 0.91 |
| R = 2 vs. R = 8 | 0.14 *( 5.15)* | 1.12 |  | 1.04 | 0.02 |  | 0.87 |
| R = 2 vs. R = 10 | -0.02 *(-0.71)* | 1.28 |  | 1.03 | -0.08 |  | 0.83 |
| R = 2 vs. R = 12 | 0.05 *( 2.03)* | 1.34 |  | 1.04 | -0.04 |  | 0.82 |
| R = 2 vs. R = 15 | 0.06 *( 2.46)* | 1.33 |  | 1.08 | -0.16 |  | 0.83 |
| R = 2 vs. R = 20 | 0.14 *( 5.31)* | 1.42 |  | 1.11 | -0.13 |  | 0.81 |
| **Velocity:** | [m/s] *(%*)* | [m/s] |  |  | [m/s] |  |  |
| R = 2 vs. R = 4 | 0.01 *( 2.00)* | 0.12 |  | 0.99 | 0.01 |  | 0.95 |
| R = 2 vs. R = 6 | -0.01 *(-1.47)* | 0.14 |  | 0.94 | 0.02 |  | 0.94 |
| R = 2 vs. R = 8 | 0.01 *( 2.20)* | 0.17 |  | 0.90 | 0.06 |  | 0.90 |
| R = 2 vs. R = 10 | -0.01 *(-2.39)* | 0.19 |  | 0.87 | 0.05 |  | 0.89 |
| R = 2 vs. R = 12 | -0.01 *(-1.31)* | 0.21 |  | 0.83 | 0.07 |  | 0.86 |
| R = 2 vs. R = 15 | -0.01 *(-2.39)* | 0.24 |  | 0.80 | 0.08 |  | 0.81 |
| R = 2 vs. R = 20 | 0.00 *(-0.29)* | 0.24 |  | 0.78 | 0.10 |  | 0.80 |

* Relative to R = 2

**Supplemental Table 3**

| ***In vivo*: Peak flow rate and velocity differences between 2D flow and R = 10/20/25/30 (averaged left and right carotid, averaged volunteers)** | |  |
| --- | --- | --- |
|  | Difference ± SD*  [ml/s] *(%**)* | Difference ± SD*  [m/s] *(%**)* |
| 2D scan vs. R = 10 | -3.3 ± 2.2 *(-16.1 ± 10.6)* | -0.03 ± 0.07 *(-6.2* ± 18.4*)* |
| 2D scan vs. R = 20 | -5.0 ± 2.1 *(-24.4 ± 10.4)* | -0.08 ± *0.04 (-15.6* ± 17.4*)* |
| 2D scan vs. R = 25 | -5.7 ± 2.4 *(-28.0 ± 12.0)* | -0.09 ± 0.05 *(-18.5* ± 24.3*)* |
| 2D scan vs. R = 30 | -6.6 ± 3.4 *(-32.6 ± 16.7)* | -0.12 ± *0.06 (-23.9* ± 29.1*)* |

* Difference for over all volunteers ± SD for volunteer values,
** Relative to the 2D scan

|  | |
| --- | --- |
|  |  |
|  |  |
|  |  |
|  |  |
|  |  |

**Supplemental Table 4**

| ***In vivo*: Differences between R = 10 and R = 20/25/30  (averaged left and right carotid, averaged volunteers)** | | | | | | | | | |
| --- | --- | --- | --- | --- | --- | --- | --- | --- | --- |
|  | Bland-Altman | |  | Orthogonal regression | |  | | Correlation | |
| **WSS:** | Mean difference  ± SD* [Pa] *(%**)* | LOA  ± SD* [Pa] | |  | Slope  ± SD* |  | Ρ  ± SD* | |  |
| R = 10 vs. R = 20 | -0.18 ± 0.21  *(-9.91 ± 10.93)* | 1.23 ± 0.29 | |  | 0.95 ± 0.22 |  | 0.76 ± 0.11 | |  |
| R = 10 vs. R = 25 | -0.25 ± 0.22 *(-13.44 ± 11.77)* | 1.29 ± 0.25 | |  | 0.78 ± 0.18 |  | 0.72 ± 0.10 | |  |
| R = 10 vs. R = 30 | -0.31 ± 0.23  *(-16.86 ± 12.16)* | 1.30 ± 0.25 | |  | 0.77 ± 0.22 |  | 0.71 ± 0.10 | |  |
|  |  |  | |  |  |  |  | |  |
| **Velocity:** | [m/s] *(%**)* | [m/s] | |  |  |  |  | |  |
| R = 10 vs. R = 20 | -0.03 ± 0.04  *(-8.35 ± 9.94)* | 0.16 ± 0.0 | |  | 0.90 ± 0.10 |  | 0.89 ± 0.03 | |  |
| R = 10 vs. R = 25 | -0.04 ± 0.04  *(-10.79 ± 9.64)* | 0.18 ± 0.0 | |  | 0.83 ± 0.10 |  | 0.87 ± 0.04 | |  |
| R = 10 vs. R = 30 | -0.05 ± 0.04 *(-13.97 ± 10.76)* | 0.18 ± 0.0 | |  | 0.81 ± 0.12 |  | 0.87 ± 0.03 | |  |

* Mean “mean difference” for all volunteers ± SD for volunteer values, ** Relative to R = 10
